# (*E*)-2-(4-Hy­droxy-3-meth­oxy­benzyl­idene)hydrazinecarboxamide

**DOI:** 10.1107/S1600536811024068

**Published:** 2011-06-25

**Authors:** Hoong-Kun Fun, Chin Sing Yeap, Shridhar Malladi, Arun M. Isloor

**Affiliations:** a X-ray Crystallography Unit, School of Physics, Universiti Sains Malaysia, 11800 USM, Penang, Malaysia; b Organic Chemistry Division, Department of Chemistry, National Institute of Technology-Karnataka, Surathkal, Mangalore 575 025, India

## Abstract

The asymmetric unit of the title compound, C_9_H_11_N_3_O_3_, consists of two crystallographically independent mol­ecules. Both mol­ecules are almost planar, with r.m.s. deviations of 0.107 and 0.099 Å. In the crystal, the two independent mol­ecules form a dimer with an *R*_2_^2^(8) ring motif *via* N—H⋯O hydrogen bonds. The dimers are further linked into a three-dimensional network by O—H⋯O and N—H⋯O hydrogen bonds.

## Related literature

For applications of semicarbazone derivatives, see: Warren *et al.* (1977 ▶); Chandra & Gupta (2005 ▶); Jain *et al.* (2002 ▶); Pilgram (1978 ▶); Yogeeswari *et al.* (2004 ▶). For the synthesis, see: Vogel *et al.* (1978 ▶). For the stability of the temperature controller used in the data collection, see: Cosier & Glazer (1986 ▶). For hydrogen-bond motifs, see: Bernstein *et al.* (1995 ▶).
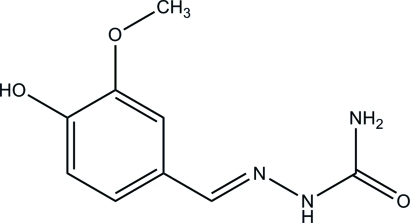


## Experimental

### 

#### Crystal data

C_9_H_11_N_3_O_3_
*M* *_r_* = 209.21
Orthorhombic, 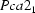

*a* = 13.8568 (3) Å
*b* = 5.0379 (1) Å
*c* = 26.8582 (5) Å
*V* = 1874.95 (7) Å^3^
*Z* = 8
Mo *K*α radiation
μ = 0.11 mm^−1^
*T* = 100 K
0.56 × 0.21 × 0.08 mm

#### Data collection

Bruker SMART APEXII CCD area-detector diffractometer
Absorption correction: multi-scan (*SADABS*; Bruker, 2009 ▶) *T*_min_ = 0.939, *T*_max_ = 0.992
27088 measured reflections
3785 independent reflections
3477 reflections with *I* > 2σ(*I*)
*R*_int_ = 0.034

#### Refinement

*R*[*F* ^2^ > 2σ(*F* ^2^)] = 0.037
*wR*(*F* ^2^) = 0.098
*S* = 1.05
3785 reflections
305 parameters
1 restraint
H atoms treated by a mixture of independent and constrained refinement
Δρ_max_ = 0.38 e Å^−3^
Δρ_min_ = −0.20 e Å^−3^

### 

Data collection: *APEX2* (Bruker, 2009 ▶); cell refinement: *SAINT* (Bruker, 2009 ▶); data reduction: *SAINT*; program(s) used to solve structure: *SHELXTL* (Sheldrick, 2008 ▶); program(s) used to refine structure: *SHELXTL*; molecular graphics: *SHELXTL*; software used to prepare material for publication: *SHELXTL* and *PLATON* (Spek, 2009 ▶).

## Supplementary Material

Crystal structure: contains datablock(s) global, I. DOI: 10.1107/S1600536811024068/is2735sup1.cif

Structure factors: contains datablock(s) I. DOI: 10.1107/S1600536811024068/is2735Isup2.hkl

Supplementary material file. DOI: 10.1107/S1600536811024068/is2735Isup3.cml

Additional supplementary materials:  crystallographic information; 3D view; checkCIF report

## Figures and Tables

**Table 1:** Hydrogen-bond geometry (Å, °)

*D*—H⋯*A*	*D*—H	H⋯*A*	*D*⋯*A*	*D*—H⋯*A*
O2*A*—H2*OA*⋯O2*B*^i^	0.86 (3)	2.22 (3)	2.9924 (18)	149 (3)
N2*A*—H2*NA*⋯O3*B*	0.88 (3)	2.03 (3)	2.8966 (19)	171 (3)
N3*A*—H3*NB*⋯O3*B*^ii^	0.89 (3)	2.10 (3)	2.961 (2)	165 (2)
N2*B*—H2*NB*⋯O3*A*	0.90 (3)	2.00 (3)	2.8812 (18)	169 (2)
N3*B*—H3*ND*⋯O3*A*^iii^	0.86 (3)	2.09 (3)	2.9415 (19)	172 (3)

Symmetry codes: (i) 
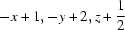
; (ii) 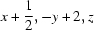; (iii) 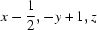.

**Fig. 1.**
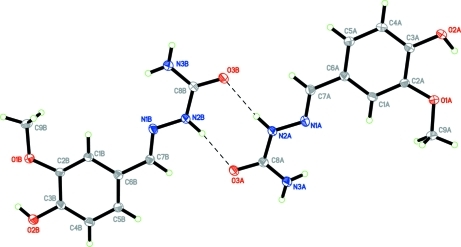
The molecular structure of the title compound, with atom labels and 50% probability ellipsoids for non-H atoms. Hydrogen bonds (dashed lines) are shown.

**Fig. 2.**
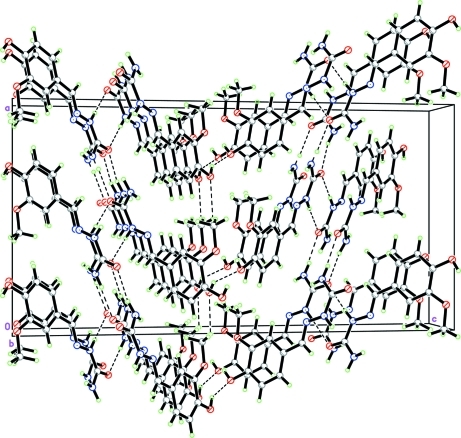
A packing diagram of the title compound viewed along the b axis, showing molecules linked into a three-dimensional network. Hydrogen bonds (dashed lines) are shown.

## supplementary crystallographic information

### Comment

In organic chemistry, semicarbazone is a derivative of an aldehyde or ketone formed by condensation between a ketone or aldehyde and a semicarbazide. Semicarbazones find a large number of applications in the field of synthetic chemistry, such as in medicinal chemistry (Warren *et al.*, 1977), organometallics (Chandra & Gupta, 2005), polymers (Jain *et al.*, 2002), and herbicides (Pilgram, 1978). 4-Sulphamoylphenyl semicarbazones were found to possess anti-convulsant activity (Yogeeswari *et al.*, 2004). Prompted by the diverse activities of semicarbazones, we have synthesized the title compound to study its crystal structure.

The asymmetric unit of title compound consists of two crystallographically independent molecules, *A* and *B* (Fig. 1). Both molecules are almost planar with the maximum deviation of 0.3177 (16) Å at N3A for molecule *A* whereas 0.1729 (12) Å at O3B for molecule *B*. The two independent molecules are interconnected by N2A—H2NA···O3B and N2B—H2NB···O3A hydrogen bonds (Fig. 1, Table 1) generating an *R*^2^_2_(8) ring motif (Bernstein *et al.*, 1995). In the crystal structure, the molecules are further linked into a three-dimensional network (Fig. 2) by O2A—H2OA···O2B, N3A—H3NB···O3B and N3B—H3ND···O3A hydrogen bonds (Table 1).

### Experimental

Semicarbazide hydrochloride (0.86 g, 7.70 mmol) and freshly re-crystallized sodium acetate (0.77 g, 9.40 mmol) were dissolved in water (10 ml) following a literature procedure (Vogel *et al.*, 1978). The reaction mixture was stirred at room temperature for 10 minutes. To this, vanillin (1.1 g, 7.23 mmol) was added and the mixture was shaken well. A little alcohol was added to dissolve the turbidity. The mixture was shaken for a further 10 minutes and allowed to stand. The title compound crystallizes on standing for 6 h. The separated crystals were filtered, washed with cold water and re-crystallized from ethanol. Yield: 1.34 g, 88.74%. *M.p.*: 502–504 K (Vogel *et al.*, 1978).

### Refinement

N-bound and O-bound hydrogen atoms were located in a difference Fourier map and refined freely. The rest of the H atoms were positioned geometrically (C—H = 0.95 or 0.98 Å) and refined using a riding model, with *U*_iso_(H) = 1.2 or 1.5*U*_eq_(C). As there are not enough anomalous dispersion to determine the absolute configuration, 2799 Friedel pairs were merged before final refinement.

### Crystal data

**Table:**

C_9_H_11_N_3_O_3_	*F*(000) = 880
*M**_r_* = 209.21	*D*_x_ = 1.482 Mg m^−^^3^
Orthorhombic, *P**c**a*2_1_	Mo *K*α radiation, λ = 0.71073 Å
Hall symbol: P 2c -2ac	Cell parameters from 8792 reflections
*a* = 13.8568 (3) Å	θ = 3.0–33.7°
*b* = 5.0379 (1) Å	µ = 0.11 mm^−^^1^
*c* = 26.8582 (5) Å	*T* = 100 K
*V* = 1874.95 (7) Å^3^	Plate, colourless
*Z* = 8	0.56 × 0.21 × 0.08 mm

### Data collection

**Table:**

Bruker SMART APEXII CCD area-detector diffractometer	3785 independent reflections
Radiation source: fine-focus sealed tube	3477 reflections with *I* > 2σ(*I*)
graphite	*R*_int_ = 0.034
φ and ω scans	θ_max_ = 33.8°, θ_min_ = 1.5°
Absorption correction: multi-scan (*SADABS*; Bruker, 2009)	*h* = −21→21
*T*_min_ = 0.939, *T*_max_ = 0.992	*k* = −7→7
27088 measured reflections	*l* = −42→34

### Refinement

**Table:**

Refinement on *F*^2^	Primary atom site location: structure-invariant direct methods
Least-squares matrix: full	Secondary atom site location: difference Fourier map
*R*[*F*^2^ > 2σ(*F*^2^)] = 0.037	Hydrogen site location: inferred from neighbouring sites
*wR*(*F*^2^) = 0.098	H atoms treated by a mixture of independent and constrained refinement
*S* = 1.05	*w* = 1/[σ^2^(*F*_o_^2^) + (0.0558*P*)^2^ + 0.2848*P*] where *P* = (*F*_o_^2^ + 2*F*_c_^2^)/3
3785 reflections	(Δ/σ)_max_ < 0.001
305 parameters	Δρ_max_ = 0.38 e Å^−^^3^
1 restraint	Δρ_min_ = −0.20 e Å^−^^3^

### Special details

**Table:**

Experimental. The crystal was placed in the cold stream of an Oxford Cryosystems Cobra open-flow nitrogen cryostat (Cosier & Glazer, 1986) operating at 100.0 (1) K.
Geometry. All e.s.d.'s (except the e.s.d. in the dihedral angle between two l.s. planes) are estimated using the full covariance matrix. The cell e.s.d.'s are taken into account individually in the estimation of e.s.d.'s in distances, angles and torsion angles; correlations between e.s.d.'s in cell parameters are only used when they are defined by crystal symmetry. An approximate (isotropic) treatment of cell e.s.d.'s is used for estimating e.s.d.'s involving l.s. planes.
Refinement. Refinement of *F*^2^ against ALL reflections. The weighted *R*-factor *wR* and goodness of fit *S* are based on *F*^2^, conventional *R*-factors *R* are based on *F*, with *F* set to zero for negative *F*^2^. The threshold expression of *F*^2^ > σ(*F*^2^) is used only for calculating *R*-factors(gt) *etc*. and is not relevant to the choice of reflections for refinement. *R*-factors based on *F*^2^ are statistically about twice as large as those based on *F*, and *R*- factors based on ALL data will be even larger.

### Fractional atomic coordinates and isotropic or equivalent isotropic displacement parameters (Å^2^)

**Table:**

	*x*	*y*	*z*	*U*_iso_*/*U*_eq_	
O1A	0.36344 (8)	1.9545 (3)	0.42045 (5)	0.0210 (2)	
O2A	0.17592 (9)	1.9564 (2)	0.43391 (5)	0.0184 (2)	
H2OA	0.220 (2)	2.071 (6)	0.4406 (12)	0.039 (8)*	
O3A	0.56955 (8)	0.7961 (2)	0.21626 (5)	0.0197 (2)	
N1A	0.42454 (10)	1.2040 (3)	0.29115 (5)	0.0167 (2)	
N2A	0.45328 (10)	0.9953 (3)	0.26135 (6)	0.0176 (3)	
H2NA	0.411 (2)	0.885 (6)	0.2484 (11)	0.034 (7)*	
N3A	0.60407 (11)	1.1886 (3)	0.25456 (6)	0.0203 (3)	
H3NA	0.581 (2)	1.323 (6)	0.2682 (12)	0.041 (8)*	
H3NB	0.6599 (18)	1.207 (5)	0.2389 (10)	0.025 (6)*	
C1A	0.35450 (11)	1.5891 (3)	0.36049 (6)	0.0156 (3)	
H1A	0.4220	1.5897	0.3544	0.019*	
C2A	0.31448 (10)	1.7697 (3)	0.39336 (6)	0.0144 (3)	
C3A	0.21404 (11)	1.7756 (3)	0.40168 (6)	0.0148 (3)	
C4A	0.15552 (11)	1.5929 (3)	0.37808 (6)	0.0173 (3)	
H4A	0.0879	1.5941	0.3839	0.021*	
C5A	0.19573 (11)	1.4062 (3)	0.34560 (6)	0.0170 (3)	
H5A	0.1553	1.2793	0.3298	0.020*	
C6A	0.29452 (11)	1.4047 (3)	0.33624 (6)	0.0152 (3)	
C7A	0.33449 (12)	1.2052 (3)	0.30250 (6)	0.0165 (3)	
H7A	0.2931	1.0740	0.2887	0.020*	
C8A	0.54501 (11)	0.9882 (3)	0.24256 (6)	0.0156 (3)	
C9A	0.46611 (11)	1.9590 (4)	0.41561 (7)	0.0213 (3)	
H9AA	0.4930	2.0937	0.4380	0.032*	
H9AB	0.4834	2.0020	0.3812	0.032*	
H9AC	0.4923	1.7845	0.4244	0.032*	
O1B	0.53465 (8)	−0.4555 (2)	0.01033 (5)	0.0185 (2)	
O2B	0.72055 (9)	−0.3736 (3)	−0.00747 (5)	0.0207 (2)	
H1OB	0.6814 (19)	−0.481 (5)	−0.0197 (11)	0.031 (7)*	
O3B	0.29971 (9)	0.6842 (2)	0.21721 (5)	0.0191 (2)	
N1B	0.44745 (10)	0.2792 (3)	0.14260 (5)	0.0161 (2)	
N2B	0.41711 (10)	0.4832 (3)	0.17293 (5)	0.0177 (3)	
H2NB	0.4597 (19)	0.600 (5)	0.1853 (10)	0.029 (6)*	
N3B	0.26476 (11)	0.2964 (3)	0.17689 (6)	0.0203 (3)	
H3ND	0.2088 (18)	0.283 (5)	0.1902 (10)	0.026 (6)*	
H3NC	0.288 (2)	0.162 (6)	0.1622 (11)	0.038 (8)*	
C1B	0.53046 (11)	−0.0990 (3)	0.07266 (6)	0.0154 (3)	
H1B	0.4640	−0.1245	0.0799	0.019*	
C2B	0.57608 (10)	−0.2581 (3)	0.03781 (6)	0.0144 (3)	
C3B	0.67512 (11)	−0.2216 (3)	0.02783 (6)	0.0156 (3)	
C4B	0.72711 (11)	−0.0285 (3)	0.05253 (6)	0.0174 (3)	
H4B	0.7940	−0.0062	0.0459	0.021*	
C5B	0.68118 (11)	0.1338 (3)	0.08729 (6)	0.0169 (3)	
H5B	0.7168	0.2673	0.1042	0.020*	
C6B	0.58328 (11)	0.1011 (3)	0.09736 (6)	0.0148 (3)	
C7B	0.53818 (11)	0.2863 (3)	0.13190 (6)	0.0157 (3)	
H7B	0.5773	0.4180	0.1472	0.019*	
C8B	0.32466 (11)	0.4926 (3)	0.19051 (6)	0.0152 (3)	
C9B	0.43242 (11)	−0.4943 (3)	0.01562 (7)	0.0192 (3)	
H9BA	0.4123	−0.6471	−0.0044	0.029*	
H9BB	0.3983	−0.3351	0.0042	0.029*	
H9BC	0.4170	−0.5272	0.0507	0.029*	

### Atomic displacement parameters (Å^2^)

**Table:**

	*U*^11^	*U*^22^	*U*^33^	*U*^12^	*U*^13^	*U*^23^
O1A	0.0126 (5)	0.0269 (6)	0.0234 (6)	−0.0001 (4)	−0.0005 (4)	−0.0095 (5)
O2A	0.0139 (5)	0.0211 (5)	0.0201 (6)	0.0010 (4)	0.0028 (4)	−0.0038 (4)
O3A	0.0163 (5)	0.0177 (5)	0.0250 (6)	−0.0002 (4)	0.0041 (4)	−0.0053 (5)
N1A	0.0182 (6)	0.0160 (5)	0.0160 (6)	0.0016 (4)	0.0019 (5)	−0.0029 (4)
N2A	0.0147 (6)	0.0173 (6)	0.0209 (6)	−0.0013 (4)	0.0041 (5)	−0.0056 (5)
N3A	0.0161 (6)	0.0185 (6)	0.0261 (7)	−0.0022 (5)	0.0036 (5)	−0.0058 (5)
C1A	0.0130 (6)	0.0177 (6)	0.0161 (6)	0.0014 (5)	0.0011 (5)	−0.0006 (5)
C2A	0.0117 (6)	0.0178 (6)	0.0137 (6)	−0.0003 (5)	−0.0007 (5)	0.0001 (5)
C3A	0.0133 (6)	0.0167 (6)	0.0144 (6)	0.0022 (5)	0.0013 (5)	0.0010 (5)
C4A	0.0122 (6)	0.0198 (6)	0.0199 (7)	0.0001 (5)	0.0015 (5)	0.0000 (5)
C5A	0.0141 (6)	0.0180 (6)	0.0188 (7)	−0.0004 (5)	−0.0001 (5)	−0.0012 (5)
C6A	0.0148 (6)	0.0161 (6)	0.0146 (6)	0.0008 (5)	0.0005 (5)	0.0000 (5)
C7A	0.0167 (6)	0.0172 (6)	0.0154 (6)	0.0011 (5)	0.0001 (5)	−0.0012 (5)
C8A	0.0150 (6)	0.0161 (6)	0.0155 (6)	0.0014 (5)	0.0000 (5)	0.0009 (5)
C9A	0.0135 (6)	0.0280 (7)	0.0223 (7)	−0.0024 (5)	0.0004 (6)	−0.0044 (6)
O1B	0.0143 (5)	0.0201 (5)	0.0212 (6)	−0.0006 (4)	0.0002 (4)	−0.0053 (4)
O2B	0.0160 (5)	0.0247 (5)	0.0213 (6)	0.0017 (4)	0.0021 (4)	−0.0074 (5)
O3B	0.0166 (5)	0.0170 (5)	0.0236 (6)	0.0011 (4)	0.0038 (4)	−0.0046 (4)
N1B	0.0168 (6)	0.0162 (5)	0.0154 (6)	0.0010 (4)	0.0024 (5)	−0.0027 (4)
N2B	0.0144 (6)	0.0181 (5)	0.0206 (6)	−0.0011 (4)	0.0038 (5)	−0.0059 (5)
N3B	0.0166 (6)	0.0186 (6)	0.0258 (7)	−0.0018 (5)	0.0032 (5)	−0.0055 (5)
C1B	0.0135 (6)	0.0177 (6)	0.0151 (6)	0.0006 (5)	0.0007 (5)	−0.0005 (5)
C2B	0.0126 (6)	0.0156 (6)	0.0151 (6)	0.0007 (5)	0.0001 (5)	−0.0004 (5)
C3B	0.0149 (6)	0.0174 (6)	0.0145 (6)	0.0028 (5)	0.0017 (5)	−0.0013 (5)
C4B	0.0135 (6)	0.0206 (6)	0.0181 (7)	0.0013 (5)	0.0012 (5)	−0.0010 (5)
C5B	0.0151 (6)	0.0190 (6)	0.0167 (7)	−0.0002 (5)	0.0002 (5)	−0.0005 (5)
C6B	0.0154 (6)	0.0160 (6)	0.0131 (6)	0.0016 (5)	0.0009 (5)	−0.0002 (5)
C7B	0.0164 (6)	0.0166 (6)	0.0140 (6)	0.0000 (5)	0.0005 (5)	−0.0008 (5)
C8B	0.0151 (6)	0.0153 (6)	0.0152 (6)	0.0011 (5)	0.0014 (5)	0.0000 (5)
C9B	0.0139 (6)	0.0232 (7)	0.0206 (7)	−0.0019 (5)	−0.0013 (5)	−0.0009 (5)

### Geometric parameters (Å, °)

**Table:**

O1A—C2A	1.3626 (19)	O1B—C2B	1.3651 (18)
O1A—C9A	1.429 (2)	O1B—C9B	1.4370 (19)
O2A—C3A	1.3630 (19)	O2B—C3B	1.3717 (19)
O2A—H2OA	0.86 (3)	O2B—H1OB	0.83 (3)
O3A—C8A	1.2458 (19)	O3B—C8B	1.2511 (18)
N1A—C7A	1.285 (2)	N1B—C7B	1.290 (2)
N1A—N2A	1.3801 (19)	N1B—N2B	1.3772 (18)
N2A—C8A	1.368 (2)	N2B—C8B	1.3661 (19)
N2A—H2NA	0.88 (3)	N2B—H2NB	0.90 (3)
N3A—C8A	1.339 (2)	N3B—C8B	1.341 (2)
N3A—H3NA	0.83 (3)	N3B—H3ND	0.86 (3)
N3A—H3NB	0.89 (3)	N3B—H3NC	0.85 (3)
C1A—C2A	1.384 (2)	C1B—C2B	1.385 (2)
C1A—C6A	1.406 (2)	C1B—C6B	1.411 (2)
C1A—H1A	0.9500	C1B—H1B	0.9500
C2A—C3A	1.410 (2)	C2B—C3B	1.410 (2)
C3A—C4A	1.381 (2)	C3B—C4B	1.380 (2)
C4A—C5A	1.399 (2)	C4B—C5B	1.395 (2)
C4A—H4A	0.9500	C4B—H4B	0.9500
C5A—C6A	1.392 (2)	C5B—C6B	1.393 (2)
C5A—H5A	0.9500	C5B—H5B	0.9500
C6A—C7A	1.462 (2)	C6B—C7B	1.457 (2)
C7A—H7A	0.9500	C7B—H7B	0.9500
C9A—H9AA	0.9800	C9B—H9BA	0.9800
C9A—H9AB	0.9800	C9B—H9BB	0.9800
C9A—H9AC	0.9800	C9B—H9BC	0.9800
			
C2A—O1A—C9A	117.26 (12)	C2B—O1B—C9B	117.40 (12)
C3A—O2A—H2OA	108 (2)	C3B—O2B—H1OB	109.6 (19)
C7A—N1A—N2A	114.92 (13)	C7B—N1B—N2B	114.11 (13)
C8A—N2A—N1A	120.12 (13)	C8B—N2B—N1B	121.10 (13)
C8A—N2A—H2NA	117.1 (19)	C8B—N2B—H2NB	117.9 (17)
N1A—N2A—H2NA	121.3 (18)	N1B—N2B—H2NB	120.4 (17)
C8A—N3A—H3NA	119 (2)	C8B—N3B—H3ND	120.2 (17)
C8A—N3A—H3NB	119.9 (17)	C8B—N3B—H3NC	118.5 (19)
H3NA—N3A—H3NB	117 (3)	H3ND—N3B—H3NC	119 (2)
C2A—C1A—C6A	119.54 (14)	C2B—C1B—C6B	119.62 (13)
C2A—C1A—H1A	120.2	C2B—C1B—H1B	120.2
C6A—C1A—H1A	120.2	C6B—C1B—H1B	120.2
O1A—C2A—C1A	126.19 (13)	O1B—C2B—C1B	126.52 (13)
O1A—C2A—C3A	113.11 (13)	O1B—C2B—C3B	113.67 (13)
C1A—C2A—C3A	120.70 (14)	C1B—C2B—C3B	119.82 (13)
O2A—C3A—C4A	120.61 (14)	O2B—C3B—C4B	119.08 (13)
O2A—C3A—C2A	119.82 (14)	O2B—C3B—C2B	120.34 (13)
C4A—C3A—C2A	119.54 (14)	C4B—C3B—C2B	120.56 (14)
C3A—C4A—C5A	120.05 (14)	C3B—C4B—C5B	119.78 (14)
C3A—C4A—H4A	120.0	C3B—C4B—H4B	120.1
C5A—C4A—H4A	120.0	C5B—C4B—H4B	120.1
C6A—C5A—C4A	120.52 (14)	C6B—C5B—C4B	120.33 (14)
C6A—C5A—H5A	119.7	C6B—C5B—H5B	119.8
C4A—C5A—H5A	119.7	C4B—C5B—H5B	119.8
C5A—C6A—C1A	119.62 (14)	C5B—C6B—C1B	119.88 (14)
C5A—C6A—C7A	119.20 (14)	C5B—C6B—C7B	117.75 (14)
C1A—C6A—C7A	121.15 (14)	C1B—C6B—C7B	122.30 (14)
N1A—C7A—C6A	121.20 (14)	N1B—C7B—C6B	122.82 (14)
N1A—C7A—H7A	119.4	N1B—C7B—H7B	118.6
C6A—C7A—H7A	119.4	C6B—C7B—H7B	118.6
O3A—C8A—N3A	123.75 (15)	O3B—C8B—N3B	123.62 (15)
O3A—C8A—N2A	118.88 (14)	O3B—C8B—N2B	118.94 (14)
N3A—C8A—N2A	117.36 (14)	N3B—C8B—N2B	117.42 (14)
O1A—C9A—H9AA	109.5	O1B—C9B—H9BA	109.5
O1A—C9A—H9AB	109.5	O1B—C9B—H9BB	109.5
H9AA—C9A—H9AB	109.5	H9BA—C9B—H9BB	109.5
O1A—C9A—H9AC	109.5	O1B—C9B—H9BC	109.5
H9AA—C9A—H9AC	109.5	H9BA—C9B—H9BC	109.5
H9AB—C9A—H9AC	109.5	H9BB—C9B—H9BC	109.5
			
C7A—N1A—N2A—C8A	173.09 (15)	C7B—N1B—N2B—C8B	−175.20 (15)
C9A—O1A—C2A—C1A	−1.7 (2)	C9B—O1B—C2B—C1B	3.9 (2)
C9A—O1A—C2A—C3A	178.26 (14)	C9B—O1B—C2B—C3B	−175.85 (14)
C6A—C1A—C2A—O1A	178.16 (15)	C6B—C1B—C2B—O1B	−178.73 (14)
C6A—C1A—C2A—C3A	−1.8 (2)	C6B—C1B—C2B—C3B	1.0 (2)
O1A—C2A—C3A—O2A	0.6 (2)	O1B—C2B—C3B—O2B	1.3 (2)
C1A—C2A—C3A—O2A	−179.49 (14)	C1B—C2B—C3B—O2B	−178.42 (14)
O1A—C2A—C3A—C4A	−177.67 (14)	O1B—C2B—C3B—C4B	179.73 (14)
C1A—C2A—C3A—C4A	2.3 (2)	C1B—C2B—C3B—C4B	0.0 (2)
O2A—C3A—C4A—C5A	−179.16 (15)	O2B—C3B—C4B—C5B	177.80 (14)
C2A—C3A—C4A—C5A	−0.9 (2)	C2B—C3B—C4B—C5B	−0.6 (2)
C3A—C4A—C5A—C6A	−0.9 (2)	C3B—C4B—C5B—C6B	0.3 (2)
C4A—C5A—C6A—C1A	1.3 (2)	C4B—C5B—C6B—C1B	0.7 (2)
C4A—C5A—C6A—C7A	179.36 (15)	C4B—C5B—C6B—C7B	−176.57 (14)
C2A—C1A—C6A—C5A	0.0 (2)	C2B—C1B—C6B—C5B	−1.3 (2)
C2A—C1A—C6A—C7A	−177.99 (15)	C2B—C1B—C6B—C7B	175.81 (14)
N2A—N1A—C7A—C6A	176.29 (14)	N2B—N1B—C7B—C6B	−175.18 (14)
C5A—C6A—C7A—N1A	176.93 (15)	C5B—C6B—C7B—N1B	177.73 (15)
C1A—C6A—C7A—N1A	−5.1 (2)	C1B—C6B—C7B—N1B	0.6 (2)
N1A—N2A—C8A—O3A	179.13 (15)	N1B—N2B—C8B—O3B	−178.70 (15)
N1A—N2A—C8A—N3A	0.4 (2)	N1B—N2B—C8B—N3B	−0.3 (2)

### Hydrogen-bond geometry (Å, °)

**Table:**

*D*—H···*A*	*D*—H	H···*A*	*D*···*A*	*D*—H···*A*
O2A—H2OA···O2B^i^	0.86 (3)	2.22 (3)	2.9924 (18)	149 (3)
N2A—H2NA···O3B	0.88 (3)	2.03 (3)	2.8966 (19)	171 (3)
N3A—H3NB···O3B^ii^	0.89 (3)	2.10 (3)	2.961 (2)	165 (2)
N2B—H2NB···O3A	0.90 (3)	2.00 (3)	2.8812 (18)	169 (2)
N3B—H3ND···O3A^iii^	0.86 (3)	2.09 (3)	2.9415 (19)	172 (3)

Symmetry codes: (i) −*x*+1, −*y*+2, *z*+1/2; (ii) *x*+1/2, −*y*+2, *z*; (iii) *x*−1/2, −*y*+1, *z*.
